# A Functional Comparison of Homopentameric Nicotinic Acetylcholine Receptors (ACR-16) Receptors From *Necator americanus* and *Ancylostoma ceylanicum*

**DOI:** 10.3389/fnmol.2020.601102

**Published:** 2020-11-26

**Authors:** Mark D. Kaji, Timothy G. Geary, Robin N. Beech

**Affiliations:** ^1^Institute of Parasitology, McGill University, Montreal, QC, Canada; ^2^School of Biological Sciences, Queen’s University-Belfast, Belfast, United Kingdom

**Keywords:** hookworms, ion channels, electrophysiology, oxantel, homology modeling, anthelmintic sensitivity, acetylcholine receptors

## Abstract

Effective control of hookworm infections in humans and animals relies on using a small group of anthelmintics. Many of these drugs target cholinergic ligand-gated ion channels, yet the direct activity of anthelmintics has only been studied in a subset of these receptors, primarily in the non-parasitic nematode, *Caenorhabditis elegans*. Here we report the characterization of a homopentameric ionotropic acetylcholine receptor (AChR), ACR-16, from *Necator americanus* and *Ancylostoma ceylanicum*, the first known characterization of human hookworm ion channels. We used two-electrode voltage clamp electrophysiology in *Xenopus laevis* oocytes to determine the pharmacodynamics of cholinergics and anthelmintics on ACR-16 from both species of hookworm. The *A. ceylanicum* receptor (Ace-ACR-16) was more sensitive to acetylcholine (EC_50_ = 20.64 ± 0.32 μM) and nicotine (EC_50_ = 24.37 ± 2.89 μM) than the *N. americanus* receptor (Nam-ACR-16) (acetylcholine EC_50_ = 170.1 ± 19.23 μM; nicotine EC_50_ = 597.9 ± 59.12 μM), at which nicotine was a weak partial agonist (% maximal acetylcholine response = 30.4 ± 7.4%). Both receptors were inhibited by 500 μM levamisole (Ace-ACR-16 = 65.1 ± 14.3% inhibition, Nam-ACR-16 = 79.5 ± 7.7% inhibition), and responded to pyrantel, but only Ace-ACR-16 responded to oxantel. We used *in silico* homology modeling to investigate potential structural differences that account for the differences in agonist binding and identified a loop E isoleucine 130 of Nam-ACR-16 as possibly playing a role in oxantel insensitivity. These data indicate that key functional differences exist among ACR-16 receptors from closely related species and suggest mechanisms for differential drug sensitivity.

## Introduction

Hookworm infection by parasites of the family *Ancylostomidae* is a source of significant morbidity in humans and companion animals, with several species capable of invading the skin of human hosts and causing an inflammatory dermal syndrome known as cutaneous larva migrans (Hochedez and Caumes, 2007). However, the vast majority of patent human infection is caused by *Necator americanus* and *Ancylostoma duodenale*, with a lesser contribution from the zoonotic parasite of dogs and cats, *Ancylostoma ceylanicum* (Hoagland and Schad, 1978; Traub, 2013). These parasites are associated with low mortality rates, but a notable longevity of infection in the intestine of their host, where they feed on blood. Untreated hookworm infections account for anemia and malnutrition responsible for large economic productivity losses (Hotez, 2008) and over 4 million social disability-adjusted life years (DALYs) lost (Bartsch et al., 2016). Furthermore, these organisms are well suited to moderate increases in temperature associated with global climate change, and represent a continuing challenge for reducing the prevalence and spread of neglected tropical diseases (Weaver et al., 2010; Okulewicz, 2017; Blum and Hotez, 2018). The severity of their social and economic impact is primarily limited through the use of anthelmintics, drugs that remove helminths from their hosts. However, there are incongruities in susceptibility to anthelmintics among the different species of hookworm. For instance, *Ancylostoma* spp. are highly sensitive to the macrocyclic lactone, ivermectin, while *N. americanus* is incompletely cleared by doses up to 25 mg/kg (Behnke et al., 1993; Tritten et al., 2012). The extent to which differences in the targets of drugs across hookworm species govern anthelmintic efficacy has not been fully investigated. This is especially important for drugs that target other soil-transmitted helminths but cannot be used against hookworm, as is the case for the cholinergic acting oxantel used to treat *Trichuris* spp. (Keiser et al., 2013). To better understand the basis of differential species responses to anthelmintics and their role in physiology, one must first fully characterize the drug targets.

Some of the most important classes of anthelmintic targets are the ionotropic acetylcholine receptors (AChRs). Nematode acetylcholine-gated cation channels are responsible for fast, excitatory neurotransmission required for a range of essential physiological functions, including movement (Touroutine et al., 2005), feeding (McKay et al., 2004), and egg laying (Duerr et al., 2001) via regulation of muscle contractions. These nematode AChRs are orthologous but pharmacologically distinct from their vertebrate host counterparts, aiding specificity for drug targeting (Atchison et al., 1992) which induces paralysis and death or expulsion from the host. Nematode AChRs include 4 subtypes, B- L- M- and N-AChRs, characterized by their subunit composition and preferential sensitivity to bephenium, levamisole, morantel and nicotine, respectively (Martin et al., 2005; Courtot et al., 2015). The major N-type AChR subunit is encoded by *acr-16*, which forms functional homopentameric receptors in the presence of the accessory protein RIC-3 (Bennett et al., 2012), and has been characterized from *C. elegans* (Ballivet et al., 1996; Raymond et al., 2000), *Ascaris suum* (Abongwa et al., 2016), *Parascaris equorum* (Charvet et al., 2018) and the dog hookworm *Ancylostoma caninum* (Choudhary et al., 2019). In *C. elegans* these receptors are expressed in body wall muscles and, along with the L-AChR, contribute to the acetylcholine response (Touroutine et al., 2005). In comparison, the *acr-16* transcript from *A. suum* has been detected in numerous tissues including the pharynx, intestine, reproductive tract as well as body wall muscles (Abongwa et al., 2016).

ACR-16 is ideal for the study of ion channel drug targets because of the growing body of function data from multiple parasitic species, the ease of expression in *Xenopus* oocytes with only a single accessory protein, and the presence of a single subunit interface type (the site of agonist binding) since it is a homopentamer. In contrast, L-AChRs exist as heteropentamers composed of 5 different subunits in *C. elegans* that are each required for expression, but different subunits combinations exist in different parasitic nematode species (Williamson et al., 2009; Neveu et al., 2010; Boulin et al., 2011; Buxton et al., 2014). This means it is not possible to be certain which subunit-subunit interfaces exist, and each combination likely has distinct binding sites. These receptors also require multiple accessory proteins for functional expression (Boulin et al., 2008, 2011; Duguet et al., 2016).

The function of ACR-16 has not been reported in hookworms that infect humans, in which recent failure of the standard treatment of the benzimidazoles albendazole or mebendazole has been documented (Humphries et al., 2011; Soukhathammavong et al., 2012), and few alternative drugs are approved for treatment. Recent work has also identified an alarming increase in treatment failure of canine hookworm infections in the United States (Castro et al., 2019). The WHO put forth a goal of reducing morbidity of helminthiases by treating 75% of human childhood hookworm infections by 2030, extended from the original 2010 and 2020 deadlines (World Health Organization, 2012, 2020). However, this goal relies solely on the use of mebendazole or albendazole, and their increasingly intensive use raises the threat of the selection and spread of drug-resistant hookworm populations. Also approved for this indication, but little used in mass drug administration campaigns for hookworm control, are pyrantel pamoate and levamisole, albeit with more limited success (Botero and Castano, 1973; Krepel et al., 1993; Reynoldson et al., 1997; Keiser and Utzinger, 2008). Oxantel pamoate is effective against whipworm infections (Howes, 1972; Garcia, 1976; Barda et al., 2018), but lacks efficacy against hookworms (Keiser et al., 2013; Speich et al., 2014; Moser et al., 2016). A combination therapy of pyrantel and oxantel has been effective in treating soil-transmitted helminth infection (Rim et al., 1975; Grandemange et al., 2007), but high level of pyrantel resistance in *A. caninum* (Kopp et al., 2007) is indicative of the threat of drug resistance.

These limitations, coupled with the fact that the global prevalence of hookworm infection has only dropped by 9% since 1996 (Hotez, 2018) despite large scale deworming programs, illustrates the urgent need to identify new safe and effective treatments to minimize the risk of rising hookworm infection prevalence. By characterizing understudied nematode ion channels, we may better understand the mechanisms by which current anthelmintics work and discover new targets to aid in the discovery of novel anthelmintics.

Here we report the identification and cloning of *acr-16* from *A. ceylanicum* and *N. americanus*, which encode functional N-type AChRs, the first ion channels to be so characterized from hookworm species that infect humans. Despite high sequence similarity between these hookworm *acr-16*, and with previously characterized nematode *acr-16*, our results identify key differences in their pharmacological profiles and suggest a structural rationale for the failure of oxantel to clear hookworm infections. These results emphasize the potential importance of species-specific drug discovery programs for hookworm infection, and perhaps for all parasitic nematodes.

## Materials and Methods

### cDNA Synthesis

Adult *A. ceylanicum* cDNA was generously provided by Dr. John Hawdon (George Washington University School of Medicine & Health Sciences, Washington, DC, United States). Adult *A. ceylanicum* and *N. americanus* were provided by the Dr. Raffi Aroian lab (University of Massachusetts Medical School, Worchester, MA, United States). Total RNA was isolated from 20 to 30 adult worms. Briefly, worms were thoroughly rinsed after removal from hamster hosts, snap frozen and crushed with a mortar and pestle until ground into a fine powder, which was suspended in TRIzol. RNA was isolated using Phenol TRIzol purification reagents and column purified with a Qiagen RNeasy MiniElute Cleanup Kit (Qiagen, Toronto, ON, United States). Quality of RNA was validated by visual inspection following electrophoresis though a denaturing agarose gel.

First strand cDNA was synthesized using a Maxima H Minus First Strand cDNA Synthesis Kit and treated with a double stranded DNase to remove genomic DNA (Thermo Fisher Scientific, Waltham, MA, United States).

### Cloning of *Ancylostoma ceylanicum* ACR-16

The full-length sequence of *A. ceylanicum* ACR-16, was predicted from a BLASTP search of *Haemonchus contortus* and *Caenorhabditis elegans* ACR-16 against the genome of *A. ceylanicum* (BioProject IDs PRJNA231479, PRJNA72583) using the web-based platform WormBase ParaSite (Howe et al., 2017). Nested PCR was performed with inner primers designed to amplify a full length, gene-specific Ace-ACR-16 transcript flanked by 5′ *Not*I and 3′ *Apa*I restriction sites added to the ends of the primers (forward outer primer 5′- GATGGAAAAGTGCACTGGGTG-3′, reverse outer primer 5′-GAGAGGAATAAGAAGAACAGACGAC-3′, inner forward primer 5′-GCGGCCGCATGTGATGCGTTCGCTGGTC-3′, in- ner reverse primer 5′-GGGCCCCACAAGGGTTAGGCGA CGAG-3′).

### Cloning of *Necator americanus* ACR-16

A partial sequence of *N. americanus* ACR-16, NECAME_12789, was obtained by BLASTP search. A primer specific for the common nematode 5′ trans-splice leader 1 (SL1) was used to amplify the 5′ end and a poly-A primer was used to amplify the 3′ end of *Nam-acr-16*. Cloned amplicons were verified Sanger sequencing (at Genome Quebec). Full length sequences were obtained from nested PCR with an inner primer flanked by 5′ *Not*I and 3′ *Apa*I restriction sites added to the ends of the primers (SL1 outer forward primer, inner forward primer 5′-ATATAGCGGCCGCATGCGTTCGTTGGTCGTCT-3′, reverse outer primer 5′-CCTCAAAAATGTCTAGAGAGTTCG-3′, inner reverse primer 5′-GGGCCCAGAGTTCGATCTAGGCG ACA-3′).

### Sequence Analysis

Signal peptide prediction was performed using the web-based program SignalP 5.0 (Nielsen and Krogh, 1998) and by sequence alignment homology using Geneious 7.17 (https://www.geneious.com). Geneious was used for all primer design [Primer3 plugin (Rozen and Skaletsky, 2000)], transmembrane domain predictions, sequence alignment and analysis (Kearse et al., 2012). Primers were synthesized by Thermo Fisher Scientific.

### cRNA Synthesis and *Xenopus laevis* Oocyte Expression

PCR products were subcloned into the pGEMT- Easy vector (Invitrogen, Waltham, MA, United States), grown using standard molecular biology techniques and plasmids were sequenced by Genome Quebec. *Ace-* and *Nam-acr-16* were cloned into the *Xenopus laevis* oocyte expression vector pTD2 (Duguet et al., 2016), a gift from Dr. T Duguet derived from pTB20 (Boulin et al., 2008). pTD2 contains a T7 promoter and the *Xenopus* 5′ and 3′ UTR of β-globin designed to improve stability of exogenous genes in the oocyte. pTD2 was linearized using *Nhe*I and *in vitro* transcription of capped copy RNA (cRNA) performed using a mMessage mMachine T7 Transcription kit according to the manufacturer’s instructions (Ambion, Burlington, ON, United States). Freshly synthesized cRNA was DNase treated, precipitated using lithium chloride and stored in nuclease- free water at −80°C.

All experiments using *X. laevis* complied with McGill University and Canadian Council on Animal Care animal protocols. Adult female *X. laevis* were purchased from Xenopus 1 (Dexter, Michigan). All surgical procedures and animal care were performed by trained personnel as outlined in AUP 2015-7758 issued by the McGill Animal Care Committee. Ovaries of *X. laevis* were surgically extracted from adult female frogs under 0.15% MS-222 tricaine methanesulphonate anesthesia (Sigma-Aldrich, Oakville, ON, United States), pH 7 corrected with sodium bicarbonate. Ovaries were cut into clumps containing roughly 15 oocytes and treated with collagenase type Ia from *Clostridium* (Sigma-Aldrich, Oakville, ON, United States) in Ca^2+^-free oocyte ringer solution (82 mM NaCl, 2 mM KCl, 1 mM MgCl_2_, 5 mM HEPES buffer, NaHCO_3_ to pH 7.3) to defolliculate and isolate individual oocytes. Post-treatment, oocytes were allowed to recover at 19°C for 1–2 h in the normal oocyte saline solution ND96 (96 mM NaCl, 2 mM KCl, 1 mM MgCl_2_, 1 mM CaCl_2_, 5 mM HEPES buffer) supplemented with pyruvate (2.5 mM) as a carbon source and penicillin (100 U/ml) and streptomycin (100 μg/ml).

### Oocyte Injections

25–50 ng of either *Ace-acr-16* or *Nam-acr-16* cRNA alone, or in equal amounts with cRNA encoding the accessory protein Hco-RIC-3 (accession # HQ116823), were loaded into mineral oil-filled borosilicate glass pipettes pulled from a P-1000 Flaming/Brown micropipette puller (Sutter Instrument Co, Novato, CA, United States) and injected into the cytoplasm of the vegetal pole of stage V or VI oocytes using a Nanoject II (Drummond Scientific Company, Broomall, PA, United States). Water injected oocytes acted as a negative control.

Oocytes were allowed a minimum of 24 h to synthesize and express receptors, then assayed daily afterward. ACR-16 is a cation channel that gates Na^+^ and Ca^2+^ ions, which can in turn activate intracellular Ca^2+^-gated Cl^–^ channels endogenous to *X. laevis* oocytes. To counteract the activity of these endogenous chloride channels, selected oocytes were incubated with 100 μM of the Ca^2+^ chelator BAPTA-AM (Sigma-Aldrich, Oakville, ON, United States), for 1 h, then washed in ND96 immediately prior to experiments.

### Drug Solutions

Unless otherwise stated, each compound was purchased from Sigma-Aldrich and dissolved in ND96 stock concentrations. Where noted, compounds were dissolved in pure DMSO and diluted in ND96 to a final concentration containing <0.1% DMSO: acetylcholine chloride, choline, betaine, (-)-nicotine hydrogen tartrate, glucosamine HCl, sodium gluconate, bephenium hydroxynaphthoate (DMSO), nornicotine, mecamylamine hydrochloride (DMSO), levamisole - (-)-tetramisole hydrochloride, pyrantel citrate (DMSO), oxantel pamoate (DMSO), morantel citrate (DMSO), ivermectin (DMSO), BAPTA-AM (DMSO).

### Electrophysiology

Two-electrode voltage clamp (TEVC) electrophysiology was used to measure the function of expressed ion channels. Briefly, oocytes were placed in a 212.5 μl (85 μl/mm, 2.5 mm tall) RC-1Z perfusion chamber (Harvard Apparatus, Saint-Laurent, QC, United States) and pierced by one voltage clamping, and one current passing electrode. Glass microelectrodes (1–5 MΩ) backfilled with 3 M KCl were connected to headstages (Axon Instruments, Foster City, CA, United States) by Ag| AgCl wires feeding into a GeneClamp 500B operational amplifier (Axon Instruments), with which user-defined holding potentials allows the measurement of changes in current across the oocyte membrane. Except for current-voltage studies, all oocytes were clamped at a holding potential of −60 mV. For current-voltage studies oocytes were subject to repeated exposures of 100 μM acetylcholine at holding potentials ranging from −75 mV to +50 mV, increasing by increments of 25 mV.

ND96 (0.1% DMSO) and the drugs used in this study were gravity-perfused into the oocyte chamber with a solution exchange rate of less than 500 ms. Once a maximal current was achieved, drug application ceased, and saline solution was restored. All agonist responses were normalized to a maximal acetylcholine response by exposing individual replicate oocytes to acetylcholine before agonist exposure. To test antagonism, compounds at indicated concentrations were co-applied to oocytes with an EC_50_ concentration of acetylcholine. For current-voltage studies a final 100 μM acetylcholine was prepared in the following solutions: ND96, 96 mM sodium gluconate, 96 mM glucosamine HCl, 1.8 mM CaCl_2_.

Recordings were digitized using Digidata 1322A (Axon Instruments). Only oocytes with intact membranes capable of maintaining voltage clamp were used.

### *In silico* Homology Modeling

Modeller v9.23 was used to generate a homology model of the extracellular domain (ECD) of ACR-16 from *A. ceylanicum* and *N. americanus*, *C. elegans*, and *Trichuris muris* (Šali and Blundell, 1993). The crystallized chimeric human α-7 extracellular domain/*Lymnaea stagnalis* acetylcholine binding protein bound to epibatidine in an open conformation (protein data bank 3SQ6) served as a template for homology modeling (Li et al., 2011). Fifty homodimer models were generated for each species of ACR-16, including the creation of models for each amino acid substitution, to recreate the binding domain between adjacent subunits, and the best models were chosen for docking simulations based on Molpdf scores and Ramachandran plot analysis calculated by Modeller.

Homodimers were used to prepare *in silico* ligand binding analysis of a single binding site between adjacent subunits, implemented by AutoDock Vina (Trott and Olson, 2010). Molecules were instructed to bind within volume of a 15 × 15 × 15 Å box encompassing the orthosteric binding site.

Fifty binding orientations were generated per root mean square from best fit with a default exhaustiveness value of 8 and the best binding poses were chosen according to predicted binding energies. All imaging was performed using USCF Chimera (Pettersen et al., 2004).

### Statistical Analysis

All statistical analyses were performed using Prism 6.0 (GraphPad Software, San Diego, CA, United States). Semi-log concentration-response curves were generated using a non-linear regression defined as:

$$\begin{array}{c} \mathrm{Imax}=\frac{1}{\left[1+\left(\frac{\mathrm{EC50}}{{\left[\text{D}\right]}^{\text{h}}}\right)\right]} \end{array}$$

where Imax is the maximal current response, [D] is the concentration of drug, EC_50_ is the value of [D] at 50% maximal response, and h is the Hill slope which was used to gauge positive cooperativity for agonist binding. No sample calculation was performed in this study.

### Ethics Statement

All experiments complied with McGill University and Canadian Council on Animal Care animal protocols. All surgical procedures and animal care were performed by trained personnel as outlined in AUP 2015-7758 issued by the McGill Animal Care Committee.

## Results

### Cloning

**FIGURE 1:**
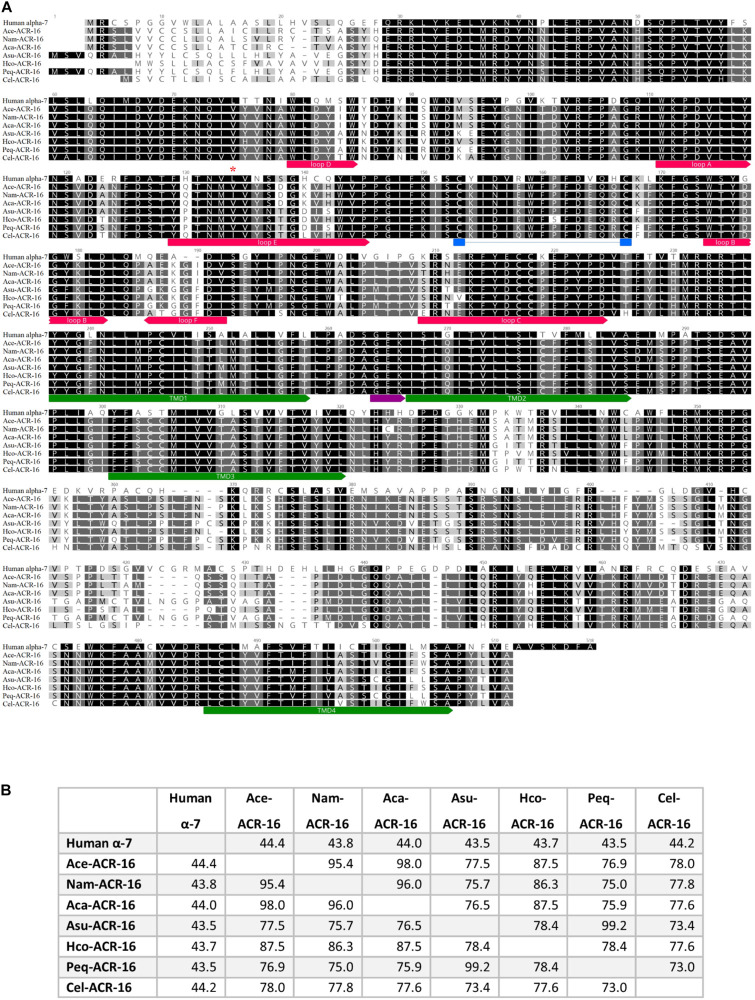
**(A)** Sequence alignment of ACR-16 from *Ancylostoma ceylanicum* (accession # MT163735), *Necator americanus* (accession # MT163736) *Ancylostoma caninum* (accession # QEM53385.1), *Caenorhabditis elegans* (accession # CCD64102.1), *Haemonchus contortus* (accession # AZS27833.1), *Ascaris suum* (accession # KP756901), *Parascaris equorum* (accession # AZS27834.1), and the human α7 acetylcholine receptor subunit (accession # P36544.5). Amino acids are shaded by consensus sequence similarity; black is most, and white is least similar. The ECD ligand binding loops A-E are denoted in red, the characteristics cys-loop is indicated in blue, the cation selectivity motif is shown in purple and the transmembrane domains are in green. Red star indicates site of Ile130 of Nam-ACR-16 **(B)**% Identity matrix of the polypeptide sequences for comparison.

Nested PCR of *Ace-* (accession # MT163735) and *Nam-acr-16* (accession # MT163736) each generated transcripts encoding 498 amino acid polypeptides. The sequence alignment (Figure 1) illustrates that both subunits contain signature pentameric ligand-gated ion channel (pLGIC) cation characteristics including a motif for cation selectivity, presence of a predicted cys-loop, 4 transmembrane domain regions, and a predicted signal peptide cleavage site after residue 21.

The amino acid sequences of Nam- and Ace-ACR-16 share 75–78% identity with ACR-16 from the clade III nematodes *A. suum* and *P. equorum*, as well as the clade V free-living nematode *C. elegans* (Meldal et al., 2007). The greatest degree of shared identity was among the hookworm ACR-16 receptors (95–98%) and the closely related Hco-ACR-16 (84–88%). Ace- and Nam-ACR-16 differ in amino acids at 22 residues, primarily within the putative signal peptide and in the C-terminal region. The *A. ceylanicum* and *A. caninum* receptors differ in 8 residues, half of which are located in the signal peptide. Regions of highest similarity include the transmembrane domains and the large extracellular domain; however, some differences exist in the aromatic loop regions that comprise the orthosteric ligand binding domain and could play a role in differential ligand specificity.

**FIGURE 2:**
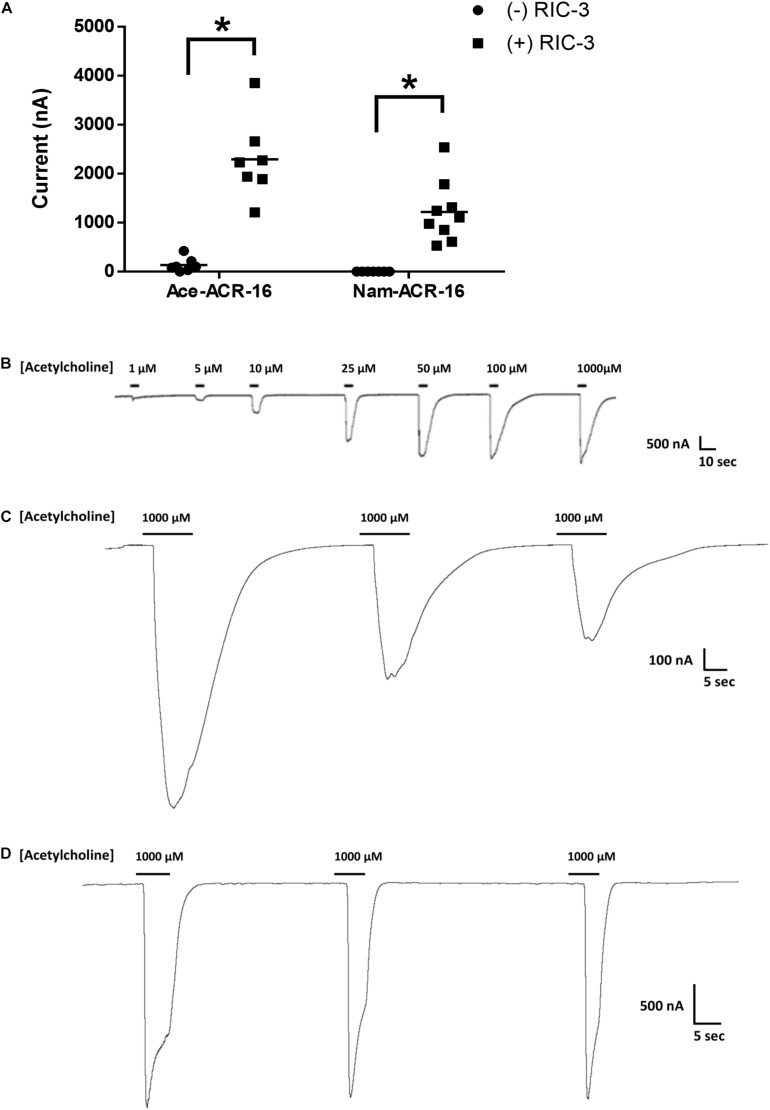
Functional expression of ACR-16 receptors in response to acetylcholine. **(A)** Amplitude of current response to 1 mM acetylcholine in the presence or absence of the accessory protein RIC-3 48 h after injection. Each point represents recordings from individual oocytes. *n* > 7; *p* < 0.05. **(B)** Ace-ACR-16 response profile to increasing concentrations of acetylcholine. **(C)** Reproducibility of Nam-ACR-16 and **(D)** Ace-ACR-16 current response profile to repeated concentrations of acetylcholine. Nam-ACR-16 displays reduced current responses to repeated exposures to acetylcholine.

The human hookworm ACR-16 receptors expressed relatively quickly, producing currents on a timescale and magnitude similar to those reported for other ACR-16s (Raymond et al., 2000; Abongwa et al., 2016; Charvet et al., 2018; Choudhary et al., 2019). Maximal responses were detected 48 hr post-cRNA injection and currents were detectable until oocyte quality degraded to the point of loss of membrane integrity, roughly 4–5 days after injection. Oocytes expressing Ace-ACR-16 alone elicited very small, but detectable current responses upon an initial 1 mM acetylcholine application, used to screen for functional expression (Figure 2A).

Oocytes co-injected with *Ace-acr-16* and *H. contortus ric-3* elicited large concentration-dependent currents in response to acetylcholine as early as 24 h after injection (Figure 2B), while oocytes injected with water produced no response. In comparison, formation of detectable Nam-ACR-16 responses required the presence of RIC-3 (Figure 2A), but with some notable and surprising differences. Nam-ACR-16 required 48 h for functional expression, the magnitude of the acetylcholine response was significantly smaller than for Ace-ACR-16, and the signal from repeated exposures to acetylcholine diminished over time (Figure 2C), in contrast to responses in Ace-ACR-16 injected oocytes, which remained constant (Figure 2D). These differences were consistent regardless of the amount of *Nam-acr-16* cRNA injected.

### Time Between Agonist Exposures

**FIGURE 3:**
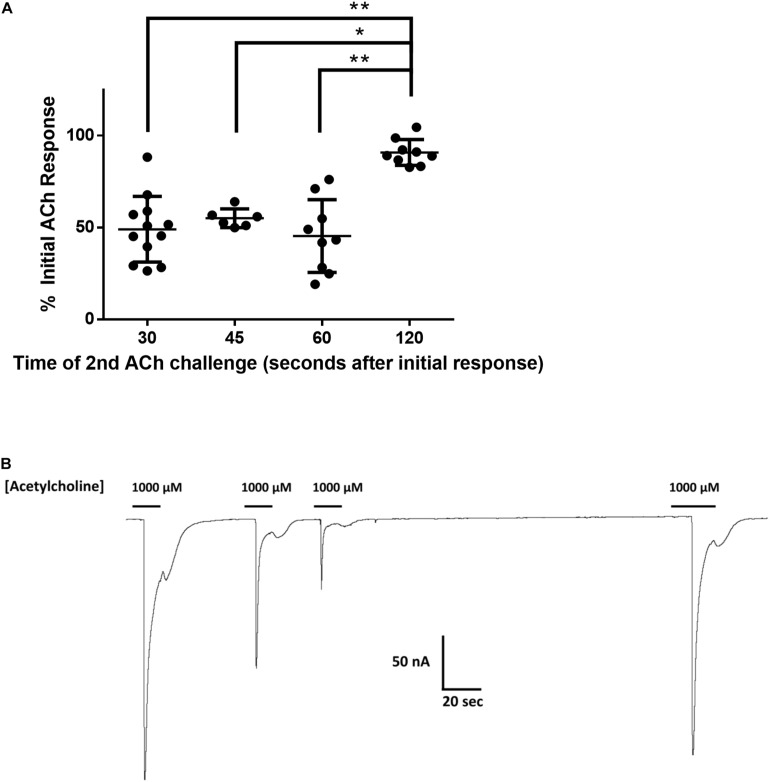
**(A)** The effect of time between subsequent applications of 1 mM acetylcholine on the magnitude of current elicited from Nam-ACR-16. Oocytes given 2 min recovery time from an initial acetylcholine exposure produced significantly larger currents than any other timepoint. Data at each time point were derived from experiments conducted on oocytes from at least two different frogs. *n* > 6, *p = 0.0004, **p < 0.0001. **(B)** Representative tracing of current responses from oocytes expressing Nam-ACR-16 induced by varying time between exposures to acetylcholine.

To investigate the reduction in Nam-ACR-16 signal from repeated exposures to a single concentration of acetylcholine, we measured the change in current amplitude as a function of time between exposures to 1 mM acetylcholine (Figure 3). Oocytes were first allowed to recover from voltage clamp and equilibrate in ND96 buffer for 1 min before recording initial responses. Incubation time prior to first exposure to acetylcholine did not influence the magnitude of the initial response.

If agonist exposure generated a refractory desensitized state in Nam-ACR-16, lengthening the recovery time between exposures should reproduce maximal responses once a greater proportion of receptors was again primed for activation. Up to 1 min after an initial response to acetylcholine, subsequent applications generated attenuated responses, and a third application sometimes failed to generate a response. Only after 2 min continual washout with ND96 solution did we see rescue of maximal amplitude, indicating a very slow but completely reversible desensitization period.

### Pharmacology

**FIGURE 4:**
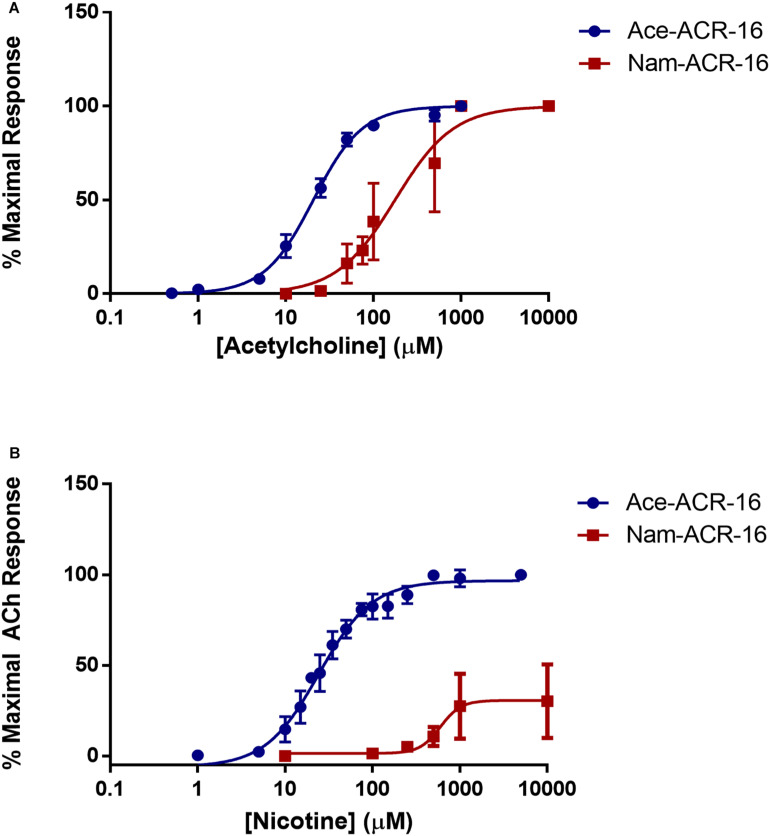
**(A)** Concentration-response curves for acetylcholine on Ace- and Nam-ACR-16. Individual oocytes were exposed to increasing concentrations of acetylcholine and all responses were standardized to the maximal current achieved within each oocyte. *n* > 6 **(B)** Concentration-response curves for nicotine on Ace- and Nam-ACR-16. Individual oocytes were exposed to repeated maximal concentrations of acetylcholine to determine stability of response, and to serve as a maximal effect reference to standardize all nicotine current responses to. Nicotine acts as a full agonist on Ace-ACR-16 but as a weak partial agonist of Nam-ACR-16 *n* > 6.

Beyond the differences reported above, we found that acetylcholine was more potent on Ace-ACR-16 (EC_50_ = 20.64 ± 0.32 μM; Hill slope = 1.55 ± 0.13) than on Nam-ACR-16 (EC_50_ = 170.1 ± 1 9.23 μM; Hill slope = 1.11 ± 0.37) (Figure 4). The lower sensitivity of Nam-ACR-16 was magnified in response to nicotine, which acted as a weak partial agonist for this receptor (EC_50_ = 597.9 ± 59.12 μM; Hill slope = 6.19 ± 1.43; maximal acetylcholine response = 30.4 ± 7.4%). Initial Nam-ACR-16 nicotine trials produced such small currents that we originally suspected degradation of the drug. However, Ace-ACR-16 receptors expressed in the same week produced large and reproducible responses to nicotine, comparable to those elicited from acetylcholine (EC_50_ = 24.37 ± 2.89 μM; Hill slope = 1.43 ± 0.15) (Figure 4B).

### Current Voltage Trials

**FIGURE 5:**
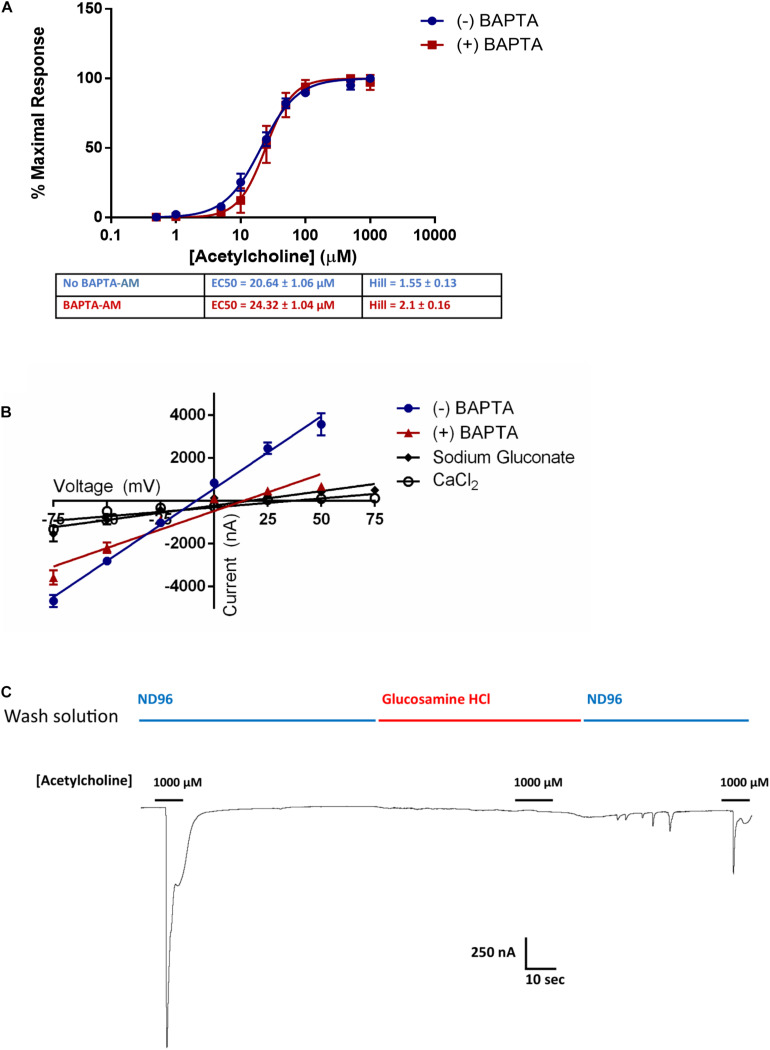
**(A)** BAPTA-AM was used to examine the role of activation of intrinsic Ca^2+^ sensing Cl^–^ channels induced by Ca^2+^ influx from Ace-ACR-16 activation. BAPTA-AM treatment had no effect on the ability of acetylcholine to activate Ace-ACR-16 in oocytes. *n* > 5; Vc = –60 mV **(B)** Current-voltage relationship of ACR-16 induced by 100 μM acetylcholine in ND96 (reversal potential = –8.5 mV), ND96 with BAPTA-treatment (reversal potential = 13.6 mV), 96 mM sodium gluconate (reversal potential = 15.9 mV), 1.8 mM CaCl_2_ (reversal potential = 35.8 mV). BAPTA-associated inhibition of Ca^2+^ gated Cl^–^ channels altered the conductance from activating ACR-16. Oocytes were exposed to 100 μM acetylcholine at holding potentials beginning at –75 mV and increasing by 25 mV to +50 mV. **(C)** 100 μM acetylcholine produced no current responses in 96 mM glucosamine HCl solution. Channel activity in response to acetylcholine was restored when this solution was replaced with ND96.

ACR-16 receptors gate Na^+^ and Ca^2+^ ions into the cell, which in turn can activate endogenous intracellular Ca^2+^-gated Cl^–^ channels in *X. laevis* oocytes (Miledi and Parker, 1984). To ensure that receptor activation measurements were not influenced by this cascading Ca^2+^ signaling, we incubated oocytes expressing Ace-ACR-16 with the intracellular Ca^2+^ ion chelator BAPTA-AM (100 μM) for 1 h. Following incubation, oocytes were washed in ND96 and voltage clamp measurements made immediately after. This receptor was chosen because the stability and reproducibility of tracings provided better accuracy than Nam-ACR-16 for comparing BAPTA-treated versus untreated oocytes. Figure 5A shows that BAPTA-AM treatment did not affect the sensitivity or activation profile of Ace-ACR-16. However, it did alter the reversal potential [(+)BAPTA-AM = 13.55 mV; (-)BAPTA-AM = -8.53 mV] and slope [(+)BAPTA-AM = 34.6 ± 2.4; (-)BAPTA-AM = 67.7 ± 2.6] of the current-voltage relationship, indicating an altered population of ions transported across the membrane (Figure 5B). Because of the variable pharmacology of ACR-16 receptors reported in the literature, we sought to validate the gating of Na^+^ and Ca^2+^ ions by completely replacing the buffer ion composition with sodium gluconate (96 mM) as an anion replacement, glucosamine HCl (96 mM) as a cation replacement or CaCl_2_ (1.8 mM). Reversal potential values from these ion replacement curves are in keeping with the values indicative of a, primarily Na^+^, cation-gated AChR (Harrow and Gration, 1985). In the absence of Na^+^ and Ca^2+^ ions in solution, no current was detectable when acetylcholine was applied (Figure 5C, glucosamine HCl).

### Panel of Anthelmintics and Classic Cholinergics Against the ACR-16 Receptors

Previous studies indicate that *Ancylostoma* spp. and *N. americanus* respond differently to anthelmintics (Behnke et al., 1993; Richards et al., 1995; Tritten et al., 2011). To investigate differences in anthelmintic sensitivity between these receptors, we tested them against a panel of drugs, including cholinergic anthelmintics.

**FIGURE 6:**
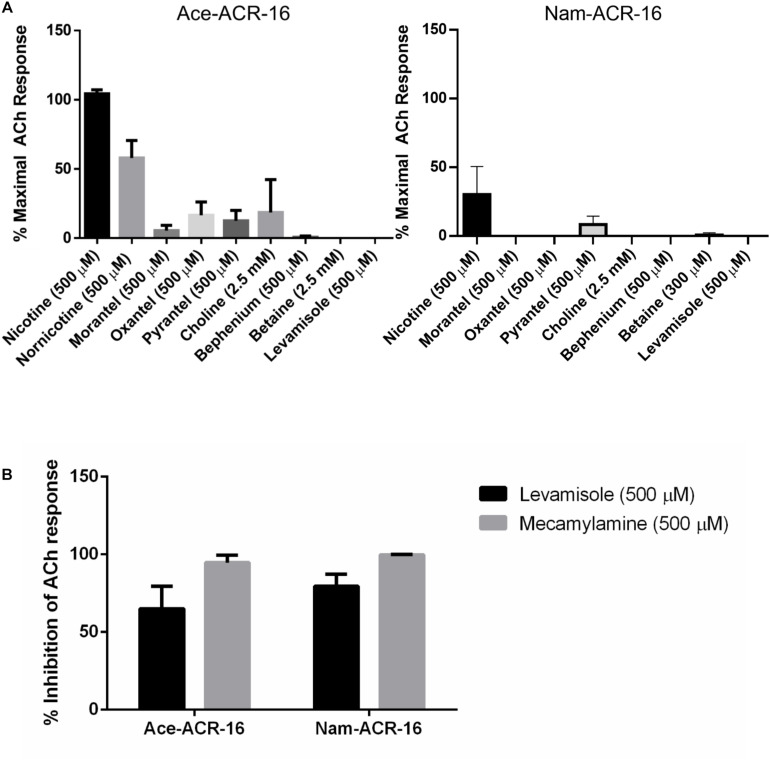
**(A)** Ace-ACR-16 (left) and Nam-ACR-16 (right) agonist response profiles to classical cholinergics and anthelmintics. All current responses are standardized relative to that of a maximal 1 mM acetylcholine response; *n* = 5 **(B)** inhibition of EC_50_ acetylcholine-induced responses in Ace- and Nam-ACR-16 by levamisole and the AChR antagonist mecamylamine; *n* ≥ 4.

Nematodes possess 4 subtypes of AChRs: nicotine (N-type), levamisole (L-type), morantel (M-type) and bephenium sensitive (B-type). As expected for an N-type AChR, neither receptor was activated by levamisole or bephenium. Ace-ACR-16 was only weakly activated by the tetrahydropyrimidines oxantel (15.6 ± 9.6% maximal acetylcholine response), pyrantel (12.5 ± 7.5%) and morantel (5.5 ± 3.9%), while Nam-ACR-16 was only activated by pyrantel (8.3 ± 6.0%) (Figure 6A). Raymond et al. (2000) showed that the *C. elegans* ACR-16 is antagonized by levamisole. To further characterize the hookworm ACR-16s, we assayed compounds and anthelmintics known to modulate the activity of pLGICs. Supporting the findings of Raymond et al. (2000), we observed significant inhibition of acetylcholine-induced channel activation by levamisole for Ace-ACR-16 (65.1 ± 14.3% inhibition) and Nam-ACR-16 (79.5 ± 7.7%) (Figure 6B). This inhibition appears to be irreversible and robust.

The classical non-competitive cholinergic antagonist mecamylamine was also inhibitory, with comparable inhibition of acetylcholine responses for Ace-ACR-16 (94.7 ± 4.7% inhibition) and Nam-ACR-16 (99.7 ± 0.4%) (Figure 6B). Ivermectin had no effect on acetylcholine-induced currents after either pre-treatment or simultaneous exposure of the oocyte.

### Homology Modeling

**FIGURE 7:**
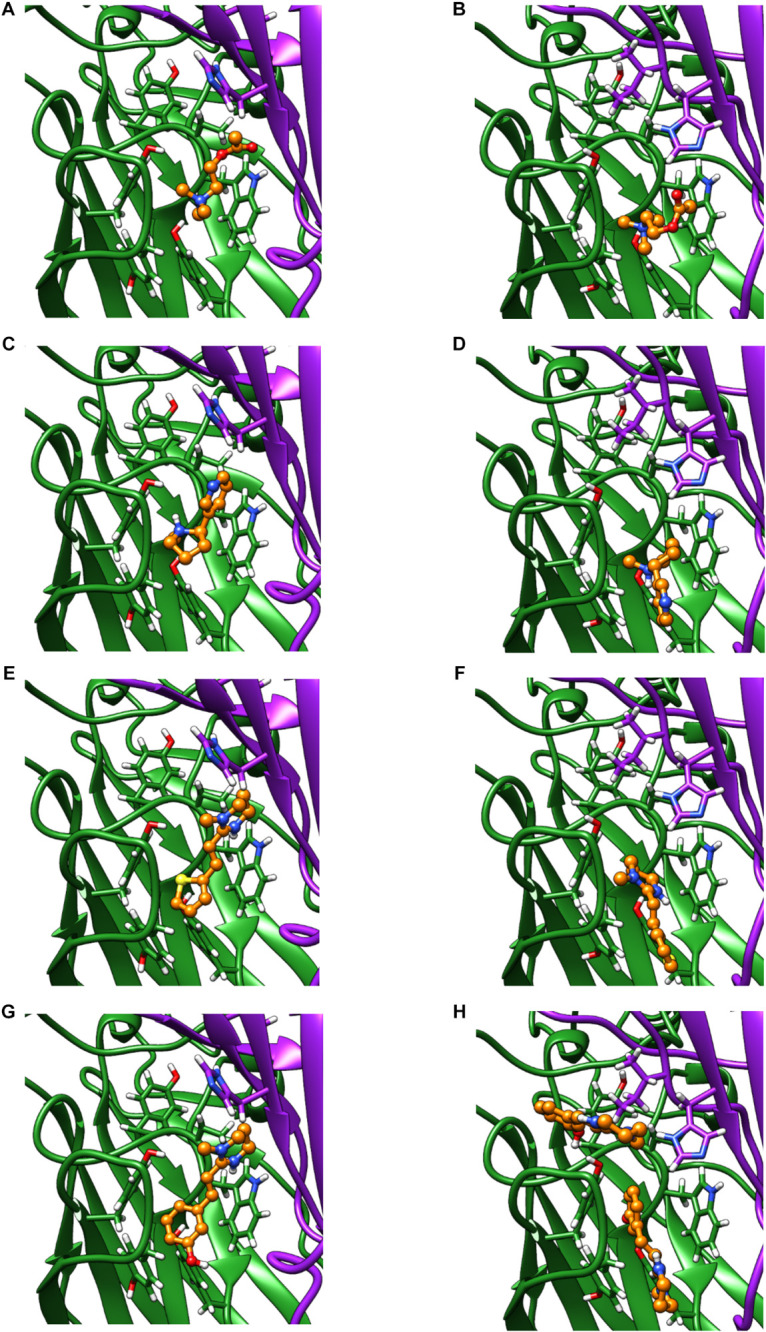
Homology models of the Ace-ACR-16 and Nam-ACR-16, docking: acetylcholine **(A,B)**, nicotine **(C,D)**, pyrantel **(E,F)**, and oxantel **(G,H)**. The principal (+) subunit contributing to key residues of Loops A, B and C, is colored green and the subunit contributing the complementary (-) face of the binding pocket contributing Loops D, E and F is colored in purple ribbon. Carbon atoms of docked agonists are colored in orange ball and stick. Red and blue molecules show oxygen and nitrogen atoms, respectively.

We used homology modeling and *in silico* ligand docking predictions to investigate structural differences between the receptors that might underlie some of the differences in pharmacology (Figure 7). Acetylcholine and nicotine docked into the orthosteric binding pocket of both ACR-16 models with comparable predicted energies (Acetylcholine: Ace-ACR-16 = −4.3 kcal/mol, Nam-ACR-16 = −4.0 kcal/mol; nicotine: Ace-ACR-16 = −5.4 kcal/mol, Nam-ACR-16 = −4.6 kcal/mol). However, docking simulations simply place the ligand in a position forming bonds with the lowest predicted binding energies, and do not take into account hydrogen bonding and π-cation interactions required for activating pLGICs.

Both agonists oriented centrally into the binding pocket of Ace-ACR-16 with their cation nitrogens placed near aromatic residues of loops B and C, notable for forming important π-cation bonds with ligands (Dougherty, 2008). In comparison, acetylcholine and nicotine docked more peripherally into Nam-ACR-16, 4–5 Å closer to the transmembrane domain, placing their cation nitrogens further from the aromatic pocket than in Ace-ACR-16. These docking differences could offer a structural origin to for the lower affinity for the site of activation in Nam-ACR-16, potentially explaining the higher EC_50_ values for this receptor. Furthermore, in the Nam-ACR-16 model, nicotine molecules did not dock with the pyridine group presenting to Loop E residues of the complementary subunit, which are expected to hydrogen bond with a water molecule essential for agonist activation (Celie et al., 2004; Blum et al., 2010). Indeed, the best scoring docking simulation for Nam-ACR-16 flipped the nitrogenous ring of nicotine away from Loop D of the complementary subunit, roughly 9 Å further than in the Ace-ACR-16 docking model. This difference conceivable prevents the complete closure of binding pocket and could explain why nicotine acted as a partial agonist on Nam-ACR-16.

Interestingly, our model also predicted significant differences in tetrahydropyrimidine (oxantel, pyrantel, morantel) binding between these receptors. When constrained to bind in the Ace-ACR-16 orthosteric agonist binding pocket, oxantel (−6.4 kcal/mol), pyrantel (−5.5 kcal/mol) and morantel (−5.7 kcal/mol) all docked with similar energies, comparable to acetylcholine and nicotine. All compounds docked within loops A-C of the aromatic box and presented functional groups to the complementary subunit where they are expected to hydrogen bond with a water molecule within the pocket. Using the same constraints for the Nam-ACR-16 receptor, all three tetrahydropyrimidine docking simulations produced positive binding energies (oxantel = + 3.6 kcal/mol; pyrantel = + 1.1 kcal/mol; morantel = + 0.7 kcal/mol) indicative of poor affinity. Only when binding parameters were extended to a large section of the extracellular domain was oxantel able to dock with a higher affinity, but still in a site and conformation peripheral to the main binding pocket. These poses either failed to present a functional group to the complementary subunit, or placed the compound above the aromatic box where they are not expected to form π-cation interactions.

**FIGURE 8:**
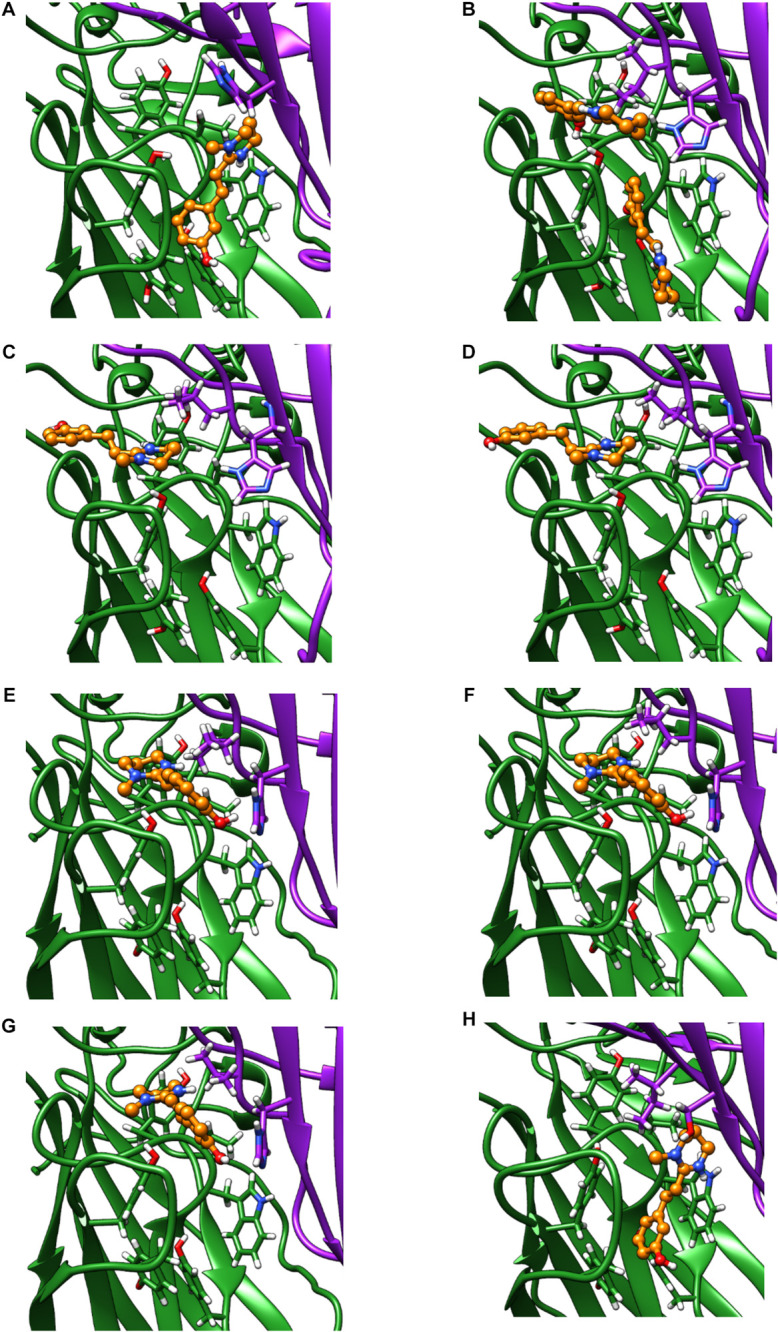
Homology models of oxantel docking into: **(A)** Ace-ACR-16 V130I **(B)** Nam-ACR-16 **(C)** Nam-ACR-16 I130L **(D)** Nam-ACR-16 I130V **(E)** Cel-ACR-16 **(F)** Cel-ACR-16 I130L **(G)** Cel-ACR-16 I130V **(H)** Tmu-ACR-16.

Only the oxantel-insensitive Cel-ACR-16 and Nam-ACR-16 possess an isoleucine on the complementary (−) subunit proximal to the binding pocket, compared to a leucine in the human α-7 receptor, and a valine in all other published ACR-16s. We modeled the Cel-ACR-16 receptor and also found an inability to dock oxantel (+2.5 kcal/mol), mirroring *in vitro* results (Raymond et al., 2000) (Figure 8). Interestingly, Ile130 in both Cel- and Nam-ACR-16 and the Val130 of the oxantel-insensitive Aca-ACR-16 points inward into the agonist binding site, whereas the side chain points in the opposite direction in Ace- and Asu-ACR-16, which both respond to oxantel. To determine if more negative space in this position is associated with improved docking predictions, we mutated the models of Nam- and Cel-ACR-16 from Ile130 to valine, which is one side-chain carbon shorter, or to a leucine, which has the same length as isoleucine but whose chain branches in the opposite direction. We then repeated the docking simulations for acetylcholine and oxantel (Figure 8) and found Val130 and Leu130 both allowed simulations to generate binding energies comparable to those of Ace-ACR-16 (Table 1), with oxantel better able to fit into the binding pocket. As expected, reverse mutation of the analogous position in Ace-ACR-16 (Val130 to isoleucine) had no effect on predicted binding parameters.

**TABLE 1:** Calculated binding energies of *in silico* simulated agonist docking.

Binding energies (kcal/mol)		
Dimer	Acetylcholine	Oxantel
Ace-ACR-16 V130→I	−4.3	−6.4
Nam-ACR-16	−4	+4.3
Nam-ACR-16 I130→L	−4.4	−3.3
Nam-ACR-16 I130→V	−4.7	−4.4
Cel-ACR-16	−3.7	+2.5
Cel-ACR-16 I130→L	−3.9	−1.5
Cel-ACR-16 I130→V	−4.2	−2
Tmu-ACR-16	−4.9	−5.4

Oxantel does not clear hookworm infections in standard single-dose regimens, but is efficacious against the whipworm *Trichuris* spp. We mined the genome of *Trichuris* spp. and, using a curated gene annotation, obtained the predicted sequences of ACR-16 from *T. muris*, *T. trichiura*, and *T. suis*. All three species possess an analogous Ile130, and our homology modeling of *T. muris* indicates that this residue extends toward the binding pocket as in Cel- and Nam-ACR-16 (Figure 8). Remarkably, docking simulations of oxantel into *Trichuris* ACR-16 yielded strong predicted binding (−5.4 kcal/mol), and in orientations more central to the binding pocket, similar to poses in Ace-ACR-16. However, the *acr-16* sequence of *Trichuris* spp. are more divergent from the clade V nematodes and contains differences in the binding pocket. One of these differences is that the predicted Tmu-ACR-16 has an outward facing V129, whereas all the other ACR-16 models have a M129 intruding into the binding pocket, which may provide more space to accommodate oxantel. Future site-directed mutagenesis studies will be required to elucidate the functional effect of these changes.

Together with the electrophysiological data, our model predictions suggest that there are separate mechanisms of binding for nicotine and acetylcholine compared to tetrahydropyrimidines, and that the Nam-ACR-16 agonist binding pocket strongly discriminates between these two classes of molecules. A caveat to the interpretation of these homology modeling results is the observation of a pyrantel-induced signal in oocytes expressing Nam-ACR-16, but no signal in response to oxantel or morantel, a difference in efficacy inconsistent with predicted binding. It is possible that the smaller and decaying nature of Nam-ACR-16 responses to agonists, compared to Ace-ACR-16, could prevent detection of low proportions of activated receptors.

## Discussion

### Expression and Agonist Activity of Acetylcholine and Nicotine

Much of our understanding of anthelmintic targets and their mechanisms of action is derived from studies of the free-living nematode, *C. elegans* (reviewed by Geary and Thompson, 2001), including the levamisole (Boulin et al., 2008) and ivermectin receptors (Cully and Paress, 1991). However, large differences in drug sensitivity have been reported between closely related parasitic nematodes, even those with high levels of sequence similarity in drug targets (Behnke et al., 1993; Tang et al., 2014; Schwarz et al., 2015). This suggests that model organisms are an appropriate starting point, but drug targets in each species of parasitic nematode must be studied individually to assess differences in pharmacodynamics. ACR-16 is one such drug target relevant for hookworms of humans and animals.

Our results indicate that the ACR-16 sequence is highly conserved across nematode species, especially the ligand-binding and transmembrane domains. With such high similarity, especially in the ligand-binding domain, one would predict very similar pharmacological profiles, and this generally holds true; Table 2 summarizes the current state of knowledge for ACR-16 receptors. As a general trend, a functional ACR-16 take 2–3 days to reach maximal expression, requires the RIC-3 accessory protein, and responds to acetylcholine and nicotine at low μM EC_50_ values. They are antagonized by the classic AChR antagonist mecamylamine and are not activated by levamisole or bephenium.

**TABLE 2:** Comparison of ACR-16 pharmacology.

Species	Acetylcholine response (μ M EC_50_)	Maximal response (current)	Nicotine response (μ M EC_50_)	Response to oxantel?	Research group
*A. ceylanicum*	20.64	nA-μA	24.33	Yes	Present study
*N. americanus*	170.1	nA	570.7	No	Present study
			*Partial agonist		
*A. caninum*	50	∼100 nA	*Partial agonist	No	Choudhary et al., 2019
*H. contortus*	Non-functional	Non-functional	Non-functional	Non-functional	Charvet et al., 2018
*C. elegans*	15.85	62.5 ± 10 nA	n/a	No	Raymond et al., 2000
*C. elegans*	55.4	nA-μA	12.6	n/a	Ballivet et al., 1996
			*Partial agonist		
*P. equorum*	6.4	μA	2.9	n/a	Charvet et al., 2018
*A. suum*	5.9	nA-μA	3.9	Yes	Abongwa et al., 2016

Despite these similarities, there are surprising differences that would not be predicted by phylogeny. The recently published *H. contortus* ACR-16 was not successfully functionally characterized, despite having high sequence identity with known functional ACR-16s (Charvet et al., 2018). An important caveat is that this protein (accession code # AZS27833.1) differs from an earlier published sequence (accession code # ABW07339.1) by a single amino acid in the cys-loop (serine versus proline in ABW07339.1). This proline residue has been reported to be of critical importance for ligand-activation in the pLGIC family (Lummis et al., 2005; Rienzo et al., 2016), and highlights the role that a single residue can play in receptor activation.

Another unexpected difference is that *A. ceylanicum* ACR-16 responses were in the large microamp range, whereas the *N. americanus* ACR-16 receptor generated currents in the nanoamp to small microamp range. Yet, the amplitude of *A. caninum* ACR-16 responses reported by Choudhary et al. (2019) better match with those from *N. americanus* rather than *A. ceylaniucm* from its own genus. Furthermore, *N. americanus* ACR-16 failed to achieve maximal channel activation from nicotine (similar to Aca- and Cel-ACR-16, but dissimilar to Ace-, Peq- and Asu-ACR-16), and nicotine was ∼fivefold less potent than acetylcholine, in contrast to the other published ACR-16s.

Surprisingly, despite being phylogenetically closer to *N. americanus* and *A. caninum*, *A. ceylanicum* ACR-16 receptor pharmacology more closely resembles that of ACR-16 from the clade III nematode *A. suum*. This is highlighted by large evoked current amplitudes, greater potency of nicotine than acetylcholine, and weak activation by oxantel on Ace- and Asu-ACR-16, but not on Nam- and Aca-ACR-16 (Abongwa et al., 2016). A limitation of our study was the use of a single, high concentration of the panel of anthelmintics to screen for agonist activity. These concentrations allowed us to compare maximal agonist activity, but did not permit the determination of competition or allosteric modulation.

### Homology Modeling

We used homology modeling to determine if structural differences in the binding pocket may explain differences in the pharmacology of *A. ceylanicum* and *N. americanus* ACR-16 receptors. Some differences in binding positions, but not binding energies of nicotine and acetylcholine were predictive of lower potency and efficacy on the Nam-ACR-16 receptor. The inability of ligands to bind deeply into the aromatic box and form hydrogen bonds with loop D of the complementary subunit has been associated with reduced channel activation for human AChRs (Celie et al., 2004; Blum et al., 2010). It is possible that the inability to dock the pyrimidine of nicotine fully into the binding pocket is related to its inability to produce maximal current responses; *in vitro* mutagenesis studies will be required to determine the binding partners involved.

Significant differences in binding energies were predicted for tetrahydropyrimidine binding, expected to occupy the same pocket (Bartos et al., 2006; Martin and Robertson, 2007). Ace-ACR-16 responded to pyrantel and oxantel *in vitro*, and both drugs docked into our *in silico* model with similar predicted energies and orientations. In contrast, Nam-ACR-16 was much less responsive to pyrantel *in vitro*, and not at all to oxantel, and neither drug could be docked into the *in silico* model with a negative kcal/mol (no bonds predicted to form). Compared to acetylcholine and nicotine, less is known regarding the intermolecular interactions required for tetrahydropyrimidine efficacy on AChRs. Pyrantel-induced activation is strongly associated with the presence of a glutamic acid in loop B (Bartos et al., 2006; Rayes et al., 2004) and a glutamine in loop D (Bartos et al., 2006). This loop D glutamine is also required for morantel, but not acetylcholine or oxantel binding to the α7 receptor (Bartos et al., 2009). Interestingly, all characterized ACR-16 subunits lack these residues, and instead possess an analogous loop D aspartic acid and loop B glycine (Positions 82 and 139 in the Figure 1 alignment, respectively). These differences in amino acid composition may explain the lack of efficacy of pyrantel and morantel, but do not explain differences between ACR-16 receptors.

Nam-ACR-16 has an isoleucine in position 130 of the (-) complementary subunit that contributes to the binding pocket. In all oxantel-insensitive ACR-16s, this analogous residue points inwards to the binding pocket, whereas it points away from the pocket in oxantel-sensitive Ace- and Asu-ACR-16 models. This difference in orientation could reflect limitations of our models, but as a counterargument it accurately predicted acetylcholine binding as in the *A. suum* ACR-16 model (Zheng et al., 2016) as well as in crystallographic analysis of acetylcholine binding (Pan et al., 2012; Olsen et al., 2014). Changing this residue to the smaller valine in either Nam- or Cel-ACR-16 allowed more space for oxantel to bind and yielded stronger predicted binding energies.

One limitation to a putative role of a smaller residue in susceptibility to oxantel is that this position is a leucine in the human α-7 AChR, for which oxantel is a weak partial agonist. Substitution of Ile130 with leucine in both Cel- and Nam-ACR-16 also rescued predicted oxantel binding, albeit in a fashion comparable to Ile130 to valine. Yao et al. (2008) showed that loop E residues play a role in neonicotinoid selectivity and may rationalize differences in Ace- and Nam-ACR-16 anthelmintic binding in our models. Another limitation to a putative role of a smaller residue in susceptibility to oxantel is that Aca-ACR-16 contains an inward facing Val130 and did not respond to oxantel. It is possible that oxantel currents are hidden by the small magnitude of current generated by this receptor, or that this extra space allows binding but not gating. Further studies using site-directed mutagenesis on these receptors may shed light on the role of the unique species-related residues involved in differential anthelmintic efficacy.

Increased space in this position alone is not likely to be a sufficient condition for determining oxantel sensitivity as the homology model of Tmu-ACR-16 also indicated the presence of an inward facing Ile130 residue, but oxantel is predicted to bind with high affinity. *Trichuris* spp. are paralyzed by oxantel, and our model of a putative *Trichuris* ACR-16 sequence also predicted strong ligand binding, despite the bulky side chain. However, the clade I *Trichuris* spp. are phylogenetically divergent from hookworms, and contain more sequence differences including the absence of a bulky histidine residue in loop E, which provides greater space for oxantel binding in our model. Interestingly, this loop E histidine is also absent in the oxantel-sensitive Asu-ACR-16 receptor (Abongwa et al., 2016).

Another unique characteristic of the putative *Trichuris* spp. ACR-16 receptors is the presence of an outward branching valine in place of a Met129 that branches into the aromatic box in the other ACR-16s. Furthermore, the human α-7 sequence also has a valine at this position but oxantel is a partial agonist on this receptor. The δ-sulfur of methionine acts as an electrophile for aromatic amino acids, forming methionine-π interactions as strong as salt bridges and up to ∼6 Å apart (Valley et al., 2012). It is possible that the presence of M129 conditionally restricts space for oxantel docking and introduces intra-subunit interactions that alter oxantel binding and activation. These differences in amino acid composition suggest that multiple species-specific residues play a role in selective oxantel sensitivity, and that Ile130 may play a secondary role in oxantel selectivity.

The relevance of differential tetrahydropyrimidine activity on ACR-16 receptors and its relationship to hookworm sensitivity to this drug class has intriguing implications. Richards et al. (1995) reported that *N. americanus* and *A. ceylanicum* are similarly paralyzed by pyrantel *in vitro*. Additionally, pyrantel and oxantel combination therapy has produced mixed reports in clearance of worms, and modest efficacy in reducing egg count in human hookworm infections (Rim et al., 1975; Moser et al., 2017). In contrast, *in vivo* studies with oxantel alone for *Ancyostoma* spp. or *N. americanus* infections indicate that it has no direct effect on the worms compared to high clearance levels against *Trichuris* spp. (Keiser et al., 2013). Interestingly, oxantel has been suggested to directly target N-type acetylcholine receptors as the mechanism of anthelmintic activity (Martin et al., 2004). If ACR-16 is the primary target of oxantel, then our results provide a mechanism to explain the lack of *in vivo* efficacy against *N. americanus*. If this is the case, then identifying a *Trichuris* spp. ACR-16 receptor and determining its *in vitro* oxantel and pyrantel sensitivity profiles may be illuminating. In support of this, the preprint of a pharmacological profile of a *T. suis* ACR-16-like receptor was made available online after the submission of this manuscript that report super-agonism by oxantel compared to acetylcholine and modest partial agonism by pyrantel (Hansen et al., 2020).

### Levamisole

In line with all published ACR-16 data, neither Ace- or Nam-ACR-16 was directly activated by levamisole, the anthelmintic activity of which is strongly associated with L-type acetylcholine receptors (Lewis et al., 1980). Of note, the first report of ACR-16 function indicated that levamisole was an antagonist of this receptor (Ballivet et al., 1996). Our results also show strong inhibition of acetylcholine responses by levamisole. ACR-16 knockout strains of *C. elegans* respond to levamisole slightly, but not significantly, less than wildtype worms (Touroutine et al., 2005); these authors did not rule out the possibility that ACR-16 sensitivity to levamisole is redundant to, and masked by, the presence of L-AChRs. When the *C. elegans* L-type AChR was abolished by a null *unc-29* allele, roughly 10% of worms were still paralyzed by exposure to levamisole (Duguet et al., 2016), suggesting a small but detectable contribution of a subset of levamisole-sensitive secondary targets. The significance of this interaction for the *in vivo* anthelmintic properties of levamisole is unclear, and we cannot discount the possibility that inhibition of ACR-16 receptors plays a role in its anthelmintic action.

### Conclusions

Our aim was to characterize ACR-16 receptors from hookworms of humans and determine differences in function attributable to species specificity. We found sensitivity to acetylcholine and nicotine, the defining features of N-type AChRs, and furthermore suggest that a structural constraint in the binding pocket of *N. americanus* accounts for the failure of oxantel to activate the receptor, which warrants further investigation. Together with existing data from ACR-16 receptors of numerous nematodes, our data suggest a great level of variability in response profiles to anthelmintics even among closely related nematode species. These implications caution against generalizing functional results of ion channel drug targets from one nematode to another and highlight the importance of relating therapeutic treatment to the unique nature of each drug target.

## Data Availability Statement

The datasets presented in this study can be found in online repositories. The names of the repository/repositories and accession number(s) can be found below: https://www.ncbi.nlm.nih.gov/, MT163735; https://www.ncbi.nlm.nih.gov/, MT163736.

## Ethics Statement

The animal study was reviewed and approved by McGill Animal Care Committee. All experiments were performed under the AUP #2015-7758 issued by the McGill Animal Care Committee.

## Author Contributions

MK designed and performed the experiments and wrote the manuscript. TG and RB supervised the project and edited the manuscript. All authors provided critical feedback to create this manuscript.

## Conflict of Interest

The authors declare that the research was conducted in the absence of any commercial or financial relationships that could be construed as a potential conflict of interest.
